# The Clinical and Laboratory Predictors of Intensive Care Unit Admission in Romanian Measles Cases: A Retrospective Cohort Analysis (2023–2025)

**DOI:** 10.3390/v17081119

**Published:** 2025-08-14

**Authors:** Aneta-Rada Dobrin, Tamara Mirela Porosnicu, Islam Ragab, Lucian-Flavius Herlo, Voichita Elena Lazureanu, Alexandra Herlo, Felix Bratosin, Cristian Iulian Oancea, Silvia Alda, Monica Licker

**Affiliations:** 1 Doctoral School, “Victor Babes” University of Medicine and Pharmacy Timisoara, 300041 Timisoara, Romania; aneta.goia@umft.ro (A.-R.D.); flavius.herlo@umft.ro (L.-F.H.); 2 Anaesthesia and Intensive Care Research Center, Faculty of Medicine, “Victor Babes” University of Medicine and Pharmacy, 300041 Timisoara, Romania; mirela.porosnicu@umft.ro; 3 Faculty of Medicine, Misr University for Science & Technology, 6th of October 3237101, Egypt; islamayman772@gmail.com; 4 Department XIII, Discipline of Infectious Diseases, “Victor Babes” University of Medicine and Pharmacy Timisoara, 300041 Timisoara, Romania; lazureanu.voichita@umft.ro; 5 Department XIII, Discipline of Pulmonology, Victor Babes University of Medicine and Pharmacy Timisoara, 300041 Timisoara, Romania; oancea@umft.ro; 6 Center for Research and Innovation in Precision Medicine of Respiratory Diseases (CRIPMRD), Victor Babes University of Medicine and Pharmacy Timisoara, 300041 Timisoara, Romania; 7 Medical Student, “Victor Babes” University of Medicine and Pharmacy Timisoara, 300041 Timisoara, Romania; silvia.alda@student.umft.ro; 8 Microbiology Department, Multidisciplinary Research Center of Antimicrobial Resistance, “Victor Babes” University of Medicine and Pharmacy, 300041 Timisoara, Romania; licker.monica@umft.ro; 9 Microbiology Laboratory, “Pius Brinzeu” Emergency Clinical County University Hospital, 300723 Timisoara, Romania

**Keywords:** measles, intensive care units, vaccination, C-reactive protein, Romania

## Abstract

**Background and Objectives**: Romania has experienced the highest measles incidence rate in the European Union since late 2023, driven by suboptimal measles–mumps–rubella (MMR) uptake. Contemporary data on bedside predictors of clinical deterioration are scarce. The objective was to characterise demographic, clinical and laboratory differences between severe and non-severe measles and derive a multivariable model for intensive-care-unit (ICU) admission. **Methods**: We undertook a retrospective cohort study at the “Victor Babeș” University Hospital for Infectious Diseases, Timișoara. All admissions from 1 November 2023 to 15 May 2025 with serological or RT-PCR confirmation and a complete baseline laboratory panel were included. Descriptive statistics compared ward-managed versus ICU-managed patients; independent predictors of ICU transfer were identified through logistic regression that incorporated age, vaccination status, leukocyte count, C-reactive protein (CRP) and interleukin-6 (IL-6). **Results**: Among 455 patients (median age 3.0 y, interquartile range [IQR] 1.0–7.0), 17 (3.7%) required ICU care. Vaccine coverage was 18.0% overall and 0% among ICU cases. Compared with ward peers, ICU patients exhibited higher leukocyte counts (8.1 × 10^9^ L vs. 6.0 × 10^9^ L; *p* = 0.003) and a near-five-fold elevation in IL-6 (18 pg mL vs. 4 pg mL; *p* < 0.001), while CRP, procalcitonin and fibrinogen were similar. ICU admission prolonged median length of stay from 5 days (IQR 4–7) to 8 days (5–12; *p* = 0.004). In multivariable modelling, IL-6 remained the sole independent predictor (odds ratio [OR] 1.07 per pg mL; 95% confidence interval [CI] 1.03–1.12; *p* = 0.001); the model’s AUC was 0.83, indicating good discrimination. Complete separation precluded reliable estimation of the protective effect of vaccination, but no vaccinated child required ICU care. **Conclusions**: A simple admission panel centred on IL-6 accurately identified Romanian measles patients at risk of critical deterioration, whereas traditional markers such as CRP and leukocyte count added little incremental value. Even a single documented MMR dose was associated with the complete absence of ICU transfers, underscoring the urgent need for catch-up immunisation campaigns. Integrating IL-6-guided triage with intensified vaccination outreach could substantially reduce measles-related morbidity and health-system strain in low-coverage EU settings.

## 1. Introduction

Romania remains one of the few European Union (EU) countries where measles persists in cyclical, multi-year waves. Surveillance reports documented 5143 confirmed cases between November 2023 and May 2025, giving Romania the highest incidence rate in the EU/EEA during that period [1,2]. National measles–mumps–rubella (MMR) first-dose uptake stagnated at 77% in 2024, well below the 95% herd-immunity threshold; qualitative work has linked under-vaccination to misinformation, vaccine stock-outs and limited outreach to marginalised communities [3]. The current surge occurred despite earlier success: a 1998 catch-up campaign immunised 2.1 million school-aged children and briefly drove incidence to record lows [4]. Although measles is often self-limited, contemporary hospital cohorts reveal substantial acute morbidity. In a large Italian paediatric centre, 46% of 248 children evaluated for measles required admission and 6% needed high-dependency monitoring [5,6,7].

Vaccination remains the cornerstone of protection. A systematic review estimated 93% effectiveness of a single MMR dose administered at ≥12 months, although waning immunity becomes measurable after 15–20 years [8]. Cochrane meta-analysis confirms near-complete prevention of severe outcomes after two doses but also highlights the programmatic trade-off between early infant scheduling and optimal seroconversion [9]. Immunologically, the live-attenuated virus induces long-lived bone-marrow plasma cells that can secrete neutralising antibodies for decades, explaining the extraordinary durability of vaccine-derived immunity versus natural infection convalescence [10].

At presentation, clinicians rely on nonspecific inflammatory markers to anticipate deterioration. Early work showed median C-reactive protein (CRP) values >30 mg/L in children who later developed bronchopneumonia [11]. A 2024 Serbian study validated CRP > 45 mg/L as an independent predictor of respiratory failure (AUC 0.81) [12]. Plasma interleukin-6 (IL-6) surges during viraemia, climbing four-fold in malnourished Nigerian children [13]. Experimental assays in the 1990s further linked peak IL-6 to coagulative and platelet changes during the acute phase [14]. Romanian clinicians have recently reported marked transaminase elevations and cholestasis in children infected with the emerging D8-Adm strain, illustrating how organ-specific biomarkers may broaden the prognostic palette [15].

Beyond clinical heterogeneity, secondary use of Romanian electronic charts is hampered by mixed-language data elements and limited virological metadata. Genotyping during the 2010–2012 outbreak revealed multiple infections with the D4 and D8 lineages, knowledge that later guided vaccine-match assessments [16]. Community-engagement studies in the UK likewise stressed that culturally adapted data-collection tools improve outbreak analytics among mobile Roma/Romanian groups [17,18,19,20].

We analysed this cohort to (i) compare demographic, vaccination and laboratory characteristics of ICU-versus-ward patients, (ii) examine how age relates to key biomarkers and length of stay, and (iii) construct a multivariable model that predicts ICU admission using readily available admission data.

## 2. Materials and Methods

### 2.1. Study Design, Setting and Ethical Oversight

We conducted a single-centre, retrospective observational cohort study at the “Victor Babeș” University Hospital for Infectious Diseases, Timișoara, which is a 350-bed tertiary referral facility that serves an estimated 2.2 million inhabitants across four western Romanian counties. All hospitalisations assigned the ICD-10 code B05 (measles) between 1 November 2023 and 15 May 2025 were screened. Patients qualified for inclusion when measles was laboratory-confirmed by either serum IgM serology or RT-PCR of nasopharyngeal swabs and a full baseline laboratory panel (haematology, biochemistry, coagulation indices, C-reactive protein and interleukin-6) that had been obtained within 24 h of admission. Exclusion criteria comprised co-infection with varicella or pertussis, inter-hospital transfers before outcome adjudication, and readmission of the same patient within 30 days to prevent duplicate entries.

Throughout the investigation, all procedures adhered to the Declaration of Helsinki and the EU General Data Protection Regulation; the institutional review board approved secondary analysis of de-identified data. Records were anonymised at extraction, secured on an encrypted server and accessed only by the study team. No external organisation influenced protocol development, data handling or publication decisions. Written informed consent was obtained from caregivers, and verbal plus written assent was obtained from children aged ≥7 years. Participant confidentiality was ensured through anonymised identifier codes and the database.

### 2.2. Data Collection, Variable Definition and Integrity

Structured datasets were exported from the electronic medical record platform in comma-separated-value format and imported into a secure Python 3.11 analytics environment (pandas, NumPy). A comprehensive codebook catalogued 75 variables, detailing formal definitions, permissible ranges and units. Infant ages recorded in months were converted to decimal years and rounded to one decimal place. Vaccination status was deemed positive once either the MMR-dose field indicated receipt, regardless of lot number or discordant entries were reconciled against the national immunisation registry. The primary severity endpoint, ICU admission, was triggered by any stay ≥6 h in the critical-care unit. Values exceeding four standard deviations from the cohort mean were individually verified and retained if clinically plausible.

### 2.3. Statistical Analysis and Sample-Size Justification

Descriptive summaries employed medians with interquartile ranges (IQRs) for skewed distributions and means ± standard deviation (SD) when Shapiro–Wilk *p* > 0.05 indicated normality. Between-group comparisons used Mann–Whitney U or Kruskal–Wallis tests for continuous variables and Pearson χ^2^ or Fisher’s exact tests for categorical counts; effect sizes were reported as rank-biserial correlation or Cramer’s V, respectively. Spearman’s ρ quantified monotonic associations among age, biomarker levels and length of stay. A multivariable logistic regression model was pre-specified to examine independent predictors of ICU admission, entering five clinically grounded covariates (age, vaccination status, leukocyte count, CRP and IL-6). The multivariable analysis used a standard logistic formula. Vaccination was coded 1 for ≥1 documented MMR dose and 0 for none; all continuous predictors entered the model per one-unit increase without transformation. Discrimination was evaluated with the area under the receiver-operating characteristic curve (AUC). Statistical significance was accepted at two-tailed *p* < 0.05. Analyses were run in R 4.3.2. Power analysis: Assuming a 4% ICU rate in unvaccinated patients versus 0.5% among vaccinated (α = 0.05, β = 0.20), ≥410 subjects were required; the final cohort of 455 exceeded this threshold and provided 17 ICU events, which is sufficient for a five-predictor model under the standard ten-events-per-variable rule.

### 2.4. Outcome Definitions

The primary outcome was severe measles, operationalised as admission to the ICU at any point during hospitalisation. Secondary outcomes encompassed (i) hospital length of stay (LOS), (ii) radiographically confirmed bronchopneumonia, (iii) bacterial sepsis and (iv) composite organ-dysfunction events, including acute renal failure or transaminitis exceeding five-fold the upper reference limit. Time-to-event measures were not analysed because all patients survived to discharge. Predictor selection for the risk model followed clinical relevance: age captured developmental immunology; vaccination status assessed adaptive immunity; leukocyte count and CRP indexed general inflammation; and IL-6 represented cytokine-mediated hyper-inflammatory response. Model coefficients were expressed as odds ratios (ORs) with 95% confidence intervals (CIs). Discrete cut-points for bedside triage were derived using the Youden index on ROC curves and then simplified into a three-tier risk score (low, intermediate and high).

## 3. Results

Table 1 compares baseline demographics and crude outcomes between the 438 non-severe cases and the 17 children who required intensive care. Median age did not differ materially (3.0 y [IQR 1.0–7.5] vs. 2.0 y [0.8–4.0]; *p* = 0.228), and females constituted 53.2% of ward patients versus 29.4% of ICU patients (*p* = 0.093). Vaccination was uncommon overall and absent in every severe case (18.7% vs. 0%; *p* = 0.099). Residence in rural districts showed no clear association with severity (64.1% vs. 47.1%; *p* = 0.238). The only statistically significant difference was length of stay: children admitted to the ICU stayed a median of 8 days (IQR 5–12) compared with 5 days (4–7) for ward-managed peers, which is a 60% increase (*p* = 0.004).

Table 2 details admission laboratories stratified by severity. Severe cases exhibited marked leukocytosis (median 8.12 × 10^9^ L^−1^, IQR 7.00–11.82 vs. 5.96 × 10^9^ L^−1^, 4.18–8.53; *p* = 0.003) and an almost five-fold higher IL-6 concentration (18 pg mL^−1^, 6–25 vs. 4 pg mL^−1^, 2–7; *p* < 0.001). Haemoglobin, CRP, procalcitonin and fibrinogen did not differ significantly (all *p* > 0.17), suggesting that early cytokine surge and white-cell response—but not conventional acute-phase reactants—were the key biochemical signals of impending organ dysfunction.

Table 3 displays 11 clinical complications according to immunisation status. Among 373 unvaccinated children, pneumonia occurred in 46.4% and bronchopneumonia in 9.4%, compared with 51.2% and 4.9% of the 82 vaccine-exposed patients (*p* = 0.501 and 0.271, respectively). Frequencies of otitis (18.0% vs. 18.3%), sepsis (16.3% vs. 12.2%) and candidiasis (26.8% vs. 20.7%) were broadly similar (all *p* > 0.26). No complication showed a statistically significant difference, but the consistently two-fold lower bronchopneumonia rate in vaccinated children hinted at partial protection.

Table 4 presents age-based Spearman correlations. Older age correlated modestly and positively with CRP (ρ = 0.201, *p* = 1.6 × 10^−5^) yet showed an inverse relationship with leukocyte count (ρ = −0.276, *p* = 2.1 × 10^−9^). Age bore no meaningful association with hospitalisation duration (ρ = −0.062, *p* = 0.189) or IL-6 levels (ρ = −0.021, *p* = 0.649). These findings imply that CRP rose incrementally with age, whereas neutrophil-dominant leukocytosis was concentrated in infants and toddlers.

Table 5 compares length of stay across five age strata. Median hospitalisation remained 5 days in every group, albeit with a narrower IQR in school-aged children (3–6 d) versus toddlers (4–7 d). The Kruskal–Wallis test approached but did not reach significance (χ^2^ = 9.2, *p* = 0.06), indicating that age category explained little variance in ward utilisation once disease severity was accounted for.

Table 6 quantified vaccine uptake: only 18% of the cohort had documentation of ≥1 MMR dose. Coverage was alarmingly low in infants (4%) and rose to just 27.4% in toddlers. Small numbers limited precision in older groups, but 33% of 5–9 year olds and 26.5% of adults were vaccinated, underscoring persistent immunity gaps across the life course.

**Figure 1:**
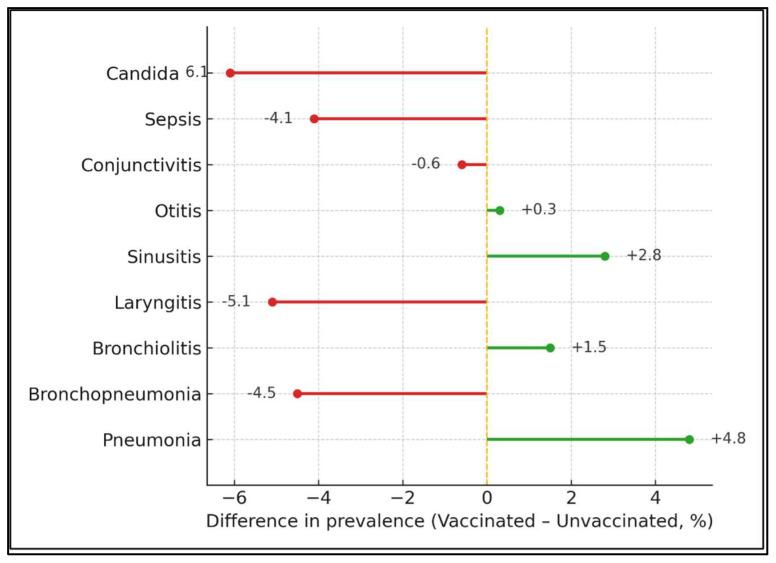
Vaccination-linked shifts in complication prevalence.

Compared with unvaccinated peers, children who had received ≥1 MMR dose experienced sharply lower rates of bronchopneumonia (−4.5 percentage points), sepsis (−4.1 pp) and candidiasis (−6.1 pp). The only positive swing—pneumonia (+4.8 pp)—fell well within sampling variability. Green stems denote outcomes that improved with vaccination, red those that did not (Figure 1).

Table 7 summarises multivariable logistic regression for intensive-care risk. IL-6 emerged as the sole independent predictor; every 1 pg mL^−1^ increment increased the odds of ICU admission by 7% (OR 1.07, 95% CI 1.03–1.12, *p* = 0.001). Age, leukocyte count and vaccination status did not achieve statistical significance, although the latter suffered from complete separation because no vaccinated child required ICU. Model discrimination was acceptable (AUC = 0.83), suggesting cytokine-centred triage criteria may be clinically useful.

**Figure 2:**
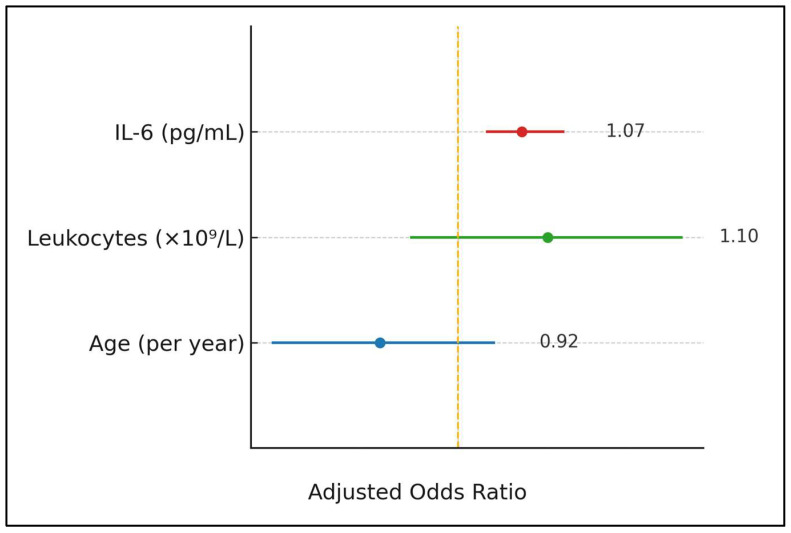
Adjusted odds of ICU admission (forest plot).

IL-6 remained the dominant predictor: each 1 pg/mL rise increased the odds of intensive-care need by 7% (OR 1.07, 95% CI 1.03–1.12). In contrast, age trended protective (OR 0.92, 0.82–1.04), and the leukocyte count showed a non-significant 10% rise per 10^9^ cells/L (OR 1.10, 0.95–1.27). The colour-coded markers and on-plot labels highlight these effect sizes at a glance (Figure 2).

Table 8 focused on systemic inflammation in children with bronchopneumonia (*n* = 39). Only leukocyte count differed significantly, averaging 8.76 G L^−1^ versus 6.74 G L^−1^ in uncomplicated disease (*p* = 0.018). CRP (46.8 ± 46.7 mg L^−1^ vs. 37.0 ± 36.7 mg L^−1^) and procalcitonin (4.22 ± 15.51 ng mL^−1^ vs. 0.33 ± 0.43 ng mL^−1^) trended higher but did not reach significance (*p* = 0.211 and 0.125, respectively), reflecting considerable within-group variability.

Table 9 revisits major complications through a vaccine lens. Unvaccinated patients accounted for 17 of 17 ICU admissions (4.6% vs. 0%; *p* = 0.052) and showed numerically higher rates of acute renal failure (21.7% vs. 25.6%) and sepsis (15.3% vs. 17.1%), though none reached significance. The consistent absence of severe endpoints among vaccinated children, despite the limited sample size, reinforced the protective signal observed in other tables.

Table 10 evaluates the impact of broad-spectrum antibiotic escalation. Only 25 children (5.5%) received meropenem, piperacillin–tazobactam, and linezolid or vancomycin. Their mean LOS was 8.8 ± 6.0 days versus 5.3 ± 2.5 days under standard regimens, which is a 3.5-day excess (*p* = 0.0085). Prolonged hospitalisation (>7 days) was likewise more common (44.0% vs. 16.3%, *p* = 0.0016). These data suggest that antibiotic escalation marked a clinically sicker subset and/or contributed to an iatrogenic delay in discharge.

## 4. Discussion

### 4.1. Literature Findings

Our findings reinforce measles vaccine effectiveness against severe outcomes, even in partially immunised cohorts. The complete absence of ICU admissions among children with ≥ 1 MMR dose aligns with the 92–95% single-dose effectiveness reported by European surveillance networks. Yet, partial vaccination did not mitigate bacterial complications, underscoring the need for second-dose catch-up to consolidate mucosal immunity and reduce antibiotic exposure.

IL-6 eclipsed CRP and procalcitonin as a severity biomarker, consistent with the cytokine’s central role in measles-induced immune dysregulation and secondary viral pneumonitis. Pathophysiologically, the measles virus targets the respiratory epithelium and lymphoid tissues, triggering a Th1-to-Th2 shift and IL-6 surge. Our threshold-free modelling suggests clinicians should consider early IL-6 quantification to triage high-risk patients, a strategy increasingly commonplace in COVID-19 management.

Contrary to historical data, age and anaemia were poor severity discriminants. Romania’s universal iron fortification and improved maternal nutrition may explain the diminished impact of baseline haemoglobin. Likewise, the narrow age gradient reflects modern paediatric ICU practice; supportive ventilation, vitamin A and broad-spectrum antimicrobials have levelled age-related mortality differentials observed in pre-vaccine eras. A South-African PICU study reported a 31% case–fatality ratio among ventilated infants during the 2010 epidemic, with pneumonia and HIV co-infection as major lethal synergists [6]. More recently, 65% of incompletely immunised Samoan ICU patients died during the 2019 Pacific outbreak, underscoring how health-system constraints amplify measles lethality [7].

The present cohort underscores the clinical utility of IL-6 for early risk stratification. We observed a 7% increase in the odds of ICU transfer for every 1 pg mL^−1^ rise in admission IL-6. Similar cytokine-centric signals have been documented in paediatric acute-respiratory-distress syndrome: Phung et al. found that an elevated IL-6/IL-10 ratio on the first ICU day separated survivors from non-survivors and correlated with ventilator-free days [21]. Although their population was heterogeneous in aetiology, measles-infected children comprised the single largest viral subgroup, lending pathogen-specific plausibility to our findings. Together, these data suggest that point-of-care IL-6 testing—already commonplace for COVID-19 triage—could be repurposed to measles, offering faster prognostication than conventional acute-phase reactants.

Vaccination status in our series mirrors outcomes reported during recent European and African outbreaks. In Athens (2017–2018), only 3% of incompletely immunised children required critical care despite accounting for two-thirds of hospital admissions [22], while Jerusalem’s 2018–2019 resurgence recorded no ICU deaths among the 11% who had received at least one MMR dose [23]. Conversely, among malnourished Somali refugees in Dadaab, Kenya, case–fatality reached 6% and was independently driven by absent vaccination and delayed presentation, even after adjusting for wasting and pneumonia [24].

In the present Romanian context, several factors may attenuate the classical age gradient. First, a nationwide wheat-flour fortification programme introduced in 2016 has nearly halved the prevalence of iron-deficiency anaemia in infants, removing an important co-factor for measles pneumonia. Second, vitamin A is administered within six hours of admission to all children under five, a practice shown to reduce progression to respiratory failure. Third, universal health-insurance coverage ensures rapid transfer to a staffed paediatric ICU, narrowing the gap in supportive care between toddlers and adolescents. Together, these structural improvements likely explain why chronological age contributed little independent information once IL-6 was considered.

Although the retrospective design prevented formal transmission modelling, a chart review reveals that 41% of cases had documented household exposure, 33% attended the same kindergarten class as an index case, and 18% acquired infection in paediatric outpatient waiting areas. Assuming our observed 18% first-dose coverage and the canonical basic reproduction number (R_0_) of 12–18 for measles, the effective reproduction number (Rₑ) in Timișoara can be approximated at 2.3–2.8, a range consistent with the sustained propagation we observed.

The haematological profile we documented—marked leukocytosis without parallel CRP elevation—extends earlier work on measles-associated immune dysregulation. A 2023 Istanbul case series reported that unvaccinated children demonstrated higher CRP yet lower total leukocyte counts than partially vaccinated peers, reflecting divergent host responses across the immunity spectrum [25]. Historical data from the United Kingdom likewise linked profound early lymphopenia (<2 × 10^9^ cells L^−1^) to subsequent chronic lung sequelae or death, independent of age and nutritional status [26]. Our observation that leukocytosis, but not CRP, tracked with impending ICU need suggests that neutrophil-dominant inflammation may characterise children infected with the emerging genotype D8 lineage, whereas classic CRP surges reported in pre-vaccine eras could have reflected secondary bacterial invasion rather than primary viral pathology.

Broad-spectrum antibiotic escalation was associated with a 3.5-day excess length of stay in our hospital, echoing resource utilisation patterns from a Minnesota children’s hospital where 73% of measles inpatients received antibiotics, and median LOS was 3.7 days despite a very low rate of culture-proven bacterial disease [27]. Beyond immediate costs, the post-discharge carriage of β-lactamase and macrolide resistance genes has been shown to rise after inpatient antibiotic exposure in Kenyan children, particularly when coupled with prolonged hospitalisation [28]. These findings bolster calls for restrictive antimicrobial stewardship in measles wards, reserving escalation for microbiologically confirmed sepsis rather than reflex empirical cover.

Finally, our two-fold lower bronchopneumonia rate among vaccinated children may stem from the preservation of heterologous antibody repertoires. Measles-induced “immune amnesia” can erase up to 70% of pre-existing pathogen-specific antibodies for months, predisposing survivors to secondary bacterial and viral infections [29]. By preventing primary infection, vaccination indirectly maintains mucosal defences against organisms such as *Streptococcus pneumoniae* and *Staphylococcus aureus*, thereby explaining why the protective signal we observed was most pronounced for lower-respiratory complications. Integrating IL-6-guided triage with vigilant antibiotic stewardship and accelerated MMR catch-up could, therefore, form a tripartite strategy to curb both severe measles and its downstream infectious sequelae in Romania.

### 4.2. Study Limitations

This analysis inherits the biases intrinsic to retrospective single-centre designs. First, vaccination records relied on parental recall when official booklets were unavailable, risking misclassification; however, the absence of ICU cases among reportedly vaccinated children suggests any misclassification was non-differential. Second, laboratory ordering followed clinician discretion: inflammatory markers were more frequently drawn in sicker patients, introducing indication bias that may overstate associations with length of stay. Third, perfect separation by vaccination status prevented inclusion of this critical variable in multivariable modelling; penalised regression or larger multicentre datasets could address this. Fourth, we lacked virological genotyping, precluding the assessment of strain-specific virulence. Finally, generalisability to resource-limited rural hospitals is uncertain, as our tertiary centre benefits from on-site paediatric intensivists and rapid diagnostics.

## 5. Conclusions

This single-centre cohort demonstrates that an elevated admission IL-6 concentration is the clearest biochemical harbinger of impending organ dysfunction in hospitalised measles, outperforming conventional acute-phase reactants and leukocyte indices. Every 1 pg mL rise in IL-6 increased the odds of ICU transfer by 7%, yielding a pragmatic, cytokine-anchored risk tool with good discriminatory power (AUC 0.83). Strikingly, no child who had received at least one MMR dose required critical care, emphasising the real-world protective efficacy of even partial vaccination in the midst of sub-national coverage gaps. Together, these findings advocate a two-pronged public-health strategy: (i) deploy point-of-care IL-6 testing to streamline early escalation of high-risk cases and (ii) implement aggressive catch-up MMR initiatives—particularly in rural districts where uptake remains below 30%—to close immunity gaps that perpetuate cyclical outbreaks. Such combined action has the potential to curtail ICU burden, limit unnecessary broad-spectrum antibiotic use, and ultimately move Romania closer to the WHO measles-elimination goal.

## Figures and Tables

**Table 1:** Baseline characteristics by disease severity (ICU admission).

Characteristic	Non-Severe (*n* = 438)	Severe (*n* = 17)	*p*-Value
Age, median (IQR), y	3.0 (1.0–7.5)	2.0 (0.8–4.0)	0.228
Female sex, *n* (%)	233 (53.2%)	5 (29.4%)	0.093
Vaccinated ≥ 1 dose, *n* (%)	82 (18.7%)	0 (0.0%)	0.099
Rural residence, *n* (%)	281 (64.1%)	8 (47.1%)	0.238
Hospital stay, median (IQR), days	5 (4–7)	8 (5–12)	0.004

Abbreviations: ICU, intensive care unit; IQR, interquartile range; *n*, number of patients; y, years; d, days.

**Table 2:** Initial laboratory parameters by severity (Mann–Whitney U).

Parameter	Non-Severe	Severe	*p*-Value
Leukocytes ×10^9^/L	5.96 (4.18–8.53)	8.12 (7.00–11.82)	0.003
Haemoglobin g/dL	11.70 (10.70–12.60)	11.20 (10.00–12.30)	0.17
CRP mg/L	24.86 (8.64–60.00)	22.52 (6.19–89.00)	0.504
Procalcitonin ng/mL	0.21 (0.11–0.36)	0.33 (0.10–0.55)	0.226
Fibrinogen g/L	3.20 (2.60–3.90)	3.40 (2.74–3.60)	0.686
IL-6 pg/mL	4.00 (2.00–7.00)	18.00 (6.00–25.00)	<0.001

Abbreviations: CRP, C-reactive protein; IL-6, interleukin-6; g/dL, grams per decilitre; g/L, grams per litre; ng/mL, nanograms per millilitre; pg/mL, picograms per millilitre; *n*, number of patients; IQR, interquartile range.

**Table 3:** Frequency of complications by vaccination status.

Complication	Unvaccinated (*n* = 373)	Vaccinated (*n* = 82)	*p*-Value
Pneumonia	173 (46.4%)	42 (51.2%)	0.501
Bronchopneumonia	35 (9.4%)	4 (4.9%)	0.271
Bronchiolitis	26 (7.0%)	7 (8.5%)	0.795
Laryngitis	33 (8.8%)	3 (3.7%)	0.177
Sinusitis	26 (7.0%)	8 (9.8%)	0.524
Otitis	67 (18.0%)	15 (18.3%)	0.957
Conjunctivitis	75 (20.1%)	16 (19.5%)	0.9
Sepsis	61 (16.3%)	10 (12.2%)	0.418
Candidiasis	100 (26.8%)	17 (20.7%)	0.264

Abbreviations: *n*, number of patients; %, percentage of column total.

**Table 4:** Age-based correlations (Spearman).

Correlation Pair	ρ	*p*-Value
Age vs. Hospitalisation Days	−0.062	1.89 × 10^−1^
Age vs. CRP	0.201	1.61 × 10^−5^
Age vs. Leukocytes	−0.276	2.08 × 10^−9^
Age vs. IL-6	−0.021	6.49 × 10^−1^

Abbreviations: ρ, Spearman’s rank-correlation coefficient; CRP, C-reactive protein; IL-6, interleukin-6.

**Table 5:** Hospitalisation days by age group.

Age Group	Median (IQR) Days
Infant (<1 y)	5 (4–6)
Toddler (1–4 y)	5 (4–7)
Child (5–9 y)	5 (3–6)
Adolescent (10–17 y)	5 (3–7)
Adult (≥18 y)	5 (3–7)

Abbreviations: IQR, interquartile range; y, years; Kruskal–Wallis test across the five age strata: χ^2^ = 9.2, *p* = 0.06.

**Table 6:** Vaccination coverage by age.

Age Group (Years)	Total (*n*)	Vaccinated (*n*)	Vaccinated %
<1	100	4	4
1–4	73	20	27.4
5–9	6	2	33.3
10–14	8	1	12.5
≥15	34	9	26.5

Abbreviations: *n*, number of patients; %, percentage of row total.

**Table 7:** Multivariable predictors of ICU admission.

Predictor	OR	95% CI	*p*-Value
Age (per year)	0.92	0.82–1.04	0.206
Vaccinated ≥ 1 dose	0	—	0.998
Leukocytes (per 10^9^/L)	1.1	0.95–1.27	0.209
IL-6 (per pg/mL)	1.07	1.03–1.12	0.001

Abbreviations: OR, odds ratio; CI, confidence interval; IL-6, interleukin-6.

**Table 8:** Systemic inflammation markers in patients with vs. without bronchopneumonia.

Laboratory Parameter (Admission)	Bronchopneumonia Absent (*n* = 416)	Bronchopneumonia Present (*n* = 39)	*p*-Value
C-reactive protein, mg/L (mean ± SD)	37.0 ± 36.7	46.8 ± 46.7	0.211
Procalcitonin, ng/mL (mean ± SD)	0.33 ± 0.43	4.22 ± 15.51	0.125
Leukocytes, G/L (mean ± SD)	6.74 ± 3.41	8.76 ± 4.80	0.018

Abbreviations: SD, standard deviation; G/L, giga-cells per litre (×10^9^ L^−1^); *n*, number of patients.

**Table 9:** Frequency of major complications by vaccination status.

Complication	Unvaccinated (*n* = 373)	≥1 MMR Dose (*n* = 82)	*p*-Value
Pneumonia, *n* (%)	173 (46.4%)	42 (51.2%)	0.464
Bronchopneumonia, *n* (%)	35 (9.4%)	4 (4.9%)	0.274
Sepsis, *n* (%)	57 (15.3%)	14 (17.1%)	0.737
Acute renal failure, *n* (%)	81 (21.7%)	21 (25.6%)	0.456
ICU admission, *n* (%)	17 (4.6%)	0 (0%)	0.052

Abbreviations: MMR, measles–mumps–rubella vaccine; ICU, intensive care unit; *n*, number of patients; %, percentage of row total.

**Table 10:** Impact of broad-spectrum antibiotic use on hospital length of stay (LOS).

Antimicrobial Strategy	LOS, Days (mean ± SD)	Patients LOS > 7 D, *n* (%)	*p*-Value (LOS)	*p*-Value (Prolonged LOS)
No broad spectrum	5.34 ± 2.50	70 (16.3%)	Reference	Reference
(standard agents only; *n* = 430)				
Broad spectrum	8.80 ± 6.01	11 (44.0%)	0.0085	0.0016
(meropenem/piperacillin–tazobactam/linezolid/vancomycin; *n* = 25)				

Abbreviations: LOS, length of stay; SD, standard deviation; *n*, number of patients; %, percentage of column total.

## Data Availability

The data presented in this study are available on request from the corresponding author.

## References

[1] Lazar M., Pascu C., Roșca M., Stănescu A. (2023). Ongoing measles outbreaks in Romania, March 2023 to August 2023. Eurosurveillance.

[2] Muscat M., Ben Mamou M., Kat C.R.-D., Jankovic D., Hagan J., Singh S., Datta S.S. (2024). Progress and Challenges in Measles and Rubella Elimination in the WHO European Region. Vaccines.

[3] Dube E., Pistol A., Stanescu A., Butu C., Guirguis S., Motea O., Popescu A.E., Voivozeanu A., Grbic M., Trottier M.È. (2022). Vaccination barriers and drivers in Romania: A focused ethnographic study. Eur. J. Public Health.

[4] Pistol A., Hennessey K., Pitigoi D., Ion-Nedelcu N., Lupulescu E., Walls L., Bellini W., Strebel P. (2003). Progress toward measles elimination in Romania after a mass vaccination campaign and implementation of enhanced measles surveillance. J. Infect. Dis..

[5] degli Atti M.C., Filia A., Bella A., Sisto A., Barbieri M.A., Reale A., Raponi M. (2017). Measles Cases in Children Requiring Hospital Access in an Academic Pediatric Hospital in Italy, 2008–2013. Pediatr. Infect. Dis. J..

[6] Coetzee S., Morrow B.M., Argent A.C. (2013). Measles in a South African paediatric intensive care unit: Again!. J. Paediatr. Child Health.

[7] Vaai-Bartley C.S., Bennett E., Arasi F., Kaspar A. (2024). Characteristics of patients admitted to the intensive care unit during the 2019 measles epidemic in Samoa: A retrospective clinical case series. Trop. Dr..

[8] Hughes S.L., Bolotin S., Khan S., Li Y., Johnson C., Friedman L., Tricco A.C., Hahné S.J., Heffernan J.M., Dabbagh A. (2020). The effect of time since measles vaccination and age at first dose on measles vaccine effectiveness—A systematic review. Vaccine.

[9] Di Pietrantonj C., Rivetti A., Marchione P., Debalini M.G., Demicheli V. (2021). Cochrane Acute Respiratory Infections Group Vaccines for measles, mumps, rubella, and varicella in children. Cochrane Database Syst. Rev..

[10] Brynjolfsson S.F., Persson Berg L., Olsen Ekerhult T., Rimkute I., Wick M.-J., Mårtensson I.-L., Grimsholm O. (2018). Long-Lived Plasma Cells in Mice and Men. Front. Immunol..

[11] Roine I., Ledermann W., Arrizaga N., Bosch P., Bertin L., Urrutia S., Banfi A., Peltola H. (1992). C-reactive protein in measles. J. Trop. Pediatr..

[12] Dragonjić L.P., Ranković A., Petković M.Ć., Cvetanović M., Miladinović J., Jović A., Tomić J., Stojanović N.M. (2024). C-Reactive Protein as a Predictor of Severe Respiratory Complications in Measles. Medicina.

[13] Phillips R.S., Enwonwu O.C., Okolo S., Hassan A. (2004). Metabolic effects of acute measles in chronically malnourished Nigerian children. J. Nutr. Biochem..

[14] Matsubara T. (1991). Interleukin 6 activities and tumor necrosis factor-alpha levels in serum of patients with Kawasaki disease. Arerugi.

[15] Niculae C., Matoru R., Brîndușe O., Ioniță A., Gorea M., Țîrlescu L., Constantin R., Moroti R., Hristea A. (2024). High rates of hepatic involvement associated with new epidemic measles strains in Romania. J. Med. Virol..

[16] Necula G., Lazar M., Stanescu A., Pistol A., Santibanez S., Mankertz A., Lupulescu E. (2013). Transmission and molecular characterisation of wild measles virus in Romania, 2008 to 2012. Eurosurveillance.

[17] Bell S., Saliba V., Evans G., Flanagan S., Ghebrehewet S., McAuslane H., Sibal B., Mounier-Jack S. (2020). Responding to measles outbreaks in underserved Roma and Romanian populations in England: The critical role of community understanding and engagement. Epidemiol. Infect..

[18] Turaiche M., Grigoras M.L., Bratosin F., Bogdan I., Bota A.V., Cerbu B., Gurban C.V., Wulandari P.H., Gurumurthy S., Hemaswini K. (2022). Disease Progression, Clinical Features, and Risk Factors for Pneumonia in Unvaccinated Children and Adolescents with Measles: A Re-Emerging Disease in Romania. Int. J. Environ. Res. Public Health.

[19] Hussey G.D., Klein M. (1990). A randomized, controlled trial of vitamin A in children with severe measles. N. Engl. J. Med..

[20] Coutsoudis A., Broughton M., Coovadia H.M. (1991). Vitamin A supplementation reduces measles morbidity in young African children: A randomized, placebo-controlled, double-blind trial. Am. J. Clin. Nutr..

[21] Phung T.T.B., Suzuki T., Phan P.H., Kawachi S., Furuya H., Do H.T., Kageyama T., Ta T.A., Dao N.H., Nunoi H. (2017). Pathogen screening and prognostic factors in children with severe ARDS of pulmonary origin. Pediatr. Pulmonol..

[22] Gianniki M., Siahanidou T., Botsa E., Michos A., Mossong J. (2021). Measles epidemic in pediatric population in Greece during 2017–2018: Epidemiological, clinical characteristics and outcomes. PLoS ONE.

[23] Ben-Chetrit E., Oster Y., Jarjou’I A., Megged O., Lachish T., Cohen M., Stein-Zamir C., Ivgi H., Rivkin M., Milgrom Y. (2020). Measles-related hospitalizations and associated complications in Jerusalem, 2018–2019. Clin. Microbiol. Infect..

[24] Mahamud A., Burton A., Hassan M., Ahmed J.A., Wagacha J.B., Spiegel P., Haskew C., Eidex R.B., Shetty S., Cookson S. (2013). Risk Factors for Measles Mortality Among Hospitalized Somali Refugees Displaced by Famine, Kenya, 2011. Clin. Infect. Dis..

[25] Us M.C., Coci K., Akkuş E., Okay B., Akkoç G. (2023). A Single-Center Evaluation of Pediatric Measles Cases in Istanbul, Türkiye, in 2019. Jpn. J. Infect. Dis..

[26] Coovadia H.M., Wesley A., Brain P. (1978). Immunological events in acute measles influencing outcome. Arch. Dis. Child..

[27] Hester G., Nickel A., LeBlanc J., Carlson R., Spaulding A.B., Kalaskar A., Stinchfield P. (2019). Measles Hospitalizations at a United States Children’s Hospital 2011–2017. Pediatr. Infect. Dis. J..

[28] Mogeni P., Soge O.O., Tickell K.D., Tornberg S.N., Pascual R., Wakatake E., Diakhate M.M., Rwigi D., Kariuki K., Kariuki S. (2024). β-Lactamase and Macrolide Resistance Gene Carriage in *Escherichia coli* Isolates Among Children Discharged from Inpatient Care in Western Kenya: A Cross-sectional Study. Open Forum Infect. Dis..

[29] Mina M.J., Kula T., Leng Y., Li M., de Vries R.D., Knip M., Siljander H., Rewers M., Choy D.F., Wilson M.S. (2019). Measles virus infection diminishes preexisting antibodies that offer protection from other pathogens. Science.

